# Genomic signatures of host‐associated divergence and adaptation in a coral‐eating snail, *Coralliophila violacea* (Kiener, 1836)

**DOI:** 10.1002/ece3.5977

**Published:** 2020-02-05

**Authors:** Sara E. Simmonds, Allison L. Fritts‐Penniman, Samantha H. Cheng, Gusti Ngurah Mahardika, Paul H. Barber

**Affiliations:** ^1^ Department of Ecology and Evolutionary Biology University of California Los Angeles Los Angeles CA USA; ^2^ Center for Biodiversity and Conservation American Museum of Natural History New York NY USA; ^3^ Animal Biomedical and Molecular Biology Laboratory Faculty of Veterinary Medicine Udayana University Bali Denpasar Indonesia

**Keywords:** adaptation, coral reefs, ecological divergence, gastropods, population genomics, RAD‐seq

## Abstract

The fluid nature of the ocean, combined with planktonic dispersal of marine larvae, lowers physical barriers to gene flow. However, divergence can still occur despite gene flow if strong selection acts on populations occupying different ecological niches. Here, we examined the population genomics of an ectoparasitic snail, *Coralliophila violacea* (Kiener 1836), that specializes on *Porites* corals in the Indo‐Pacific. Previous genetic analyses revealed two sympatric lineages associated with different coral hosts. In this study, we examined the mechanisms promoting and maintaining the snails’ adaptation to their coral hosts. Genome‐wide single nucleotide polymorphism (SNP) data from type II restriction site‐associated DNA (2b‐RAD) sequencing revealed two differentiated clusters of *C. violacea* that were largely concordant with coral host, consistent with previous genetic results. However, the presence of some admixed genotypes indicates gene flow from one lineage to the other. Combined, these results suggest that differentiation between host‐associated lineages of *C. violacea* is occurring in the face of ongoing gene flow, requiring strong selection. Indeed, 2.7% of all SNP loci were outlier loci (73/2,718), indicative of divergence with gene flow, driven by adaptation of each *C. violacea* lineage to their specific coral hosts.

## Graphical abstract

In this study, we used genome‐wide data to investigate whether the ecological divergence observed in a parasitic marine snail, *Coralliophila violacea*, that specializes on *Porites* coral occurred via directional selection on different hosts. We saw evidence of migration between hosts and found loci under divergent selection, including a gene involved in the control of xenobiotic detoxification pathway gene expression, perhaps allowing snails to neutralize coral‐specific toxins. These findings provide strong support for ecological divergence with gene flow, driven by adaptation to host.

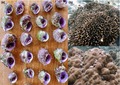

## 1 INTRODUCTION

While ecological speciation has been documented for almost three decades across a wide variety of organisms on land (Case & Willis, 2008; Feder et al., 1994; Jiggins, 2008; Martin et al., 2013; Schluter, 2009; Seehausen et al., 2008; Sorenson, Sefc, & Payne, 2003; Thorpe, Surget‐Groba, & Johansson, 2010; Waser & Campbell, 2004) and in freshwater (Hatfield & Schluter, 1999; Langerhans, Gifford, & Joseph, 2007; Puebla, 2009; Seehausen et al., 2008; Seehausen & Wagner, 2014), ecological speciation in the ocean was thought to be rare, and only recently has that viewpoint begun to change (Bird, Fernandez‐Silva, Skillings, & Toonen, 2012; Bird, Holland, Bowen, & Toonen, 2011; Bowen, Rocha, Toonen, Karl, & ToBo Laboratory, 2013; Foote & Morin, 2015; Hurt, Silliman, Anker, & Knowlton, 2013; Ingram, 2010; Litsios et al., 2012; Rocha, Robertson, Roman, & Bowen, 2005). There are a number of reasons for this reassessment. First, absolute physical barriers in the sea are exceedingly rare (Ludt & Rocha, 2015; Rocha & Bowen, 2008; Rocha et al., 2005). As a result, speciation must often proceed with varying levels of gene flow and aided by divergent selection in different habitats or hosts (Palumbi, 1994). Second, the strong interspecific interactions that can promote ecological speciation in terrestrial species (e.g., host–parasite, mutualisms) are also common in certain marine ecosystems (Blackall, Wilson, & van Oppen, 2015; Stella, Jones, & Pratchett, 2010). For example, reef‐building corals have tight ecological associations with a wide variety of invertebrate taxa (Zann, 1987), including ~900 named species of sponges, copepods, barnacles, crabs, shrimp, worms, bivalves, nudibranchs, and snails (reviewed by Stella et al., 2010). This wide array of symbiotic relationships creates tremendous potential for host shifting and the development of host specificity that can lead to sympatric speciation.

Evidence from traditional genetic markers (i.e., microsatellites, RFLPs, allozymes, nuclear, mitochondrial, and ribosomal genes) demonstrates the potential for ecological speciation in marine taxa exhibiting symbiotic relationships (Bowen et al., 2013; Miglietta, Faucci, & Santini, 2011; Peijnenburg & Goetze, 2013; Potkamp & Fransen, 2019), including amphipods on macroalgae (Sotka, 2005), coral‐dwelling barnacles (Tsang, Chan, Shih, Chu, & Allen Chen, 2009), coral‐eating nudibranchs (Faucci, Toonen, & Hadfield, 2007; Fritts‐Penniman, Gosliner, Mahardika, & Barber, 2020), parasitic snails (Gittenberger & Gittenberger, 2011; Reijnen, Hoeksema, & Gittenberger, 2010), anemone‐associated shrimp (Hurt et al., 2013), anemone fish (Litsios et al., 2012), and coral‐dwelling gobies (Duchene, Klanten, Munday, Herler, & van Herwerden, 2013; Munday, van Herwerden, & Dudgeon, 2004). While encouraging, there are gaps in our knowledge that with the expansion of genomic technologies, we are now in a position to begin to fill. Detecting signatures of natural selection in populations where there is likely ongoing gene flow is now possible using genome‐wide data, lending insight into the mechanisms of ecological speciation (Bernal, Gaither, Simison, & Rocha, 2017; Campbell, Poelstra, & Yoder, 2018; Puebla, Bermingham, & McMillan, 2014; Westram et al., 2018). To date, however, no studies examining the genomic signatures of ecological divergence in marine host–parasite systems have been conducted.

The ~6 million km^2^ Coral Triangle region is home to over 500 species of reef‐building corals (Veron et al., 2011) and thousands of unique species of fishes and invertebrates (Barber & Boyce, 2006; Briggs, 2003), making it the global center of marine biodiversity (Cowman & Bellwood, 2011; Hoeksema, 2007). Most of the literature examining the evolution of this biodiversity hotspot has focused on allopatric processes such as divergence across geological and oceanographic features such as the Sunda Shelf or Halmahera Eddy during Pleistocene low sea levels stands (for reviews, see Barber, Cheng, Erdmann, Tenggardjaja, & Ambariyanto 2011; Carpenter et al., 2011; Gaither & Rocha, 2013). Allopatric divergence is clearly an important factor in the biodiversity of the Coral Triangle. However, the extraordinary diversity in this region, combined with the prevalence of strong species–species interactions on coral reefs, makes it likely that ecological speciation also contributes to the evolution of biodiversity in this hotspot.

**Figure 1:**
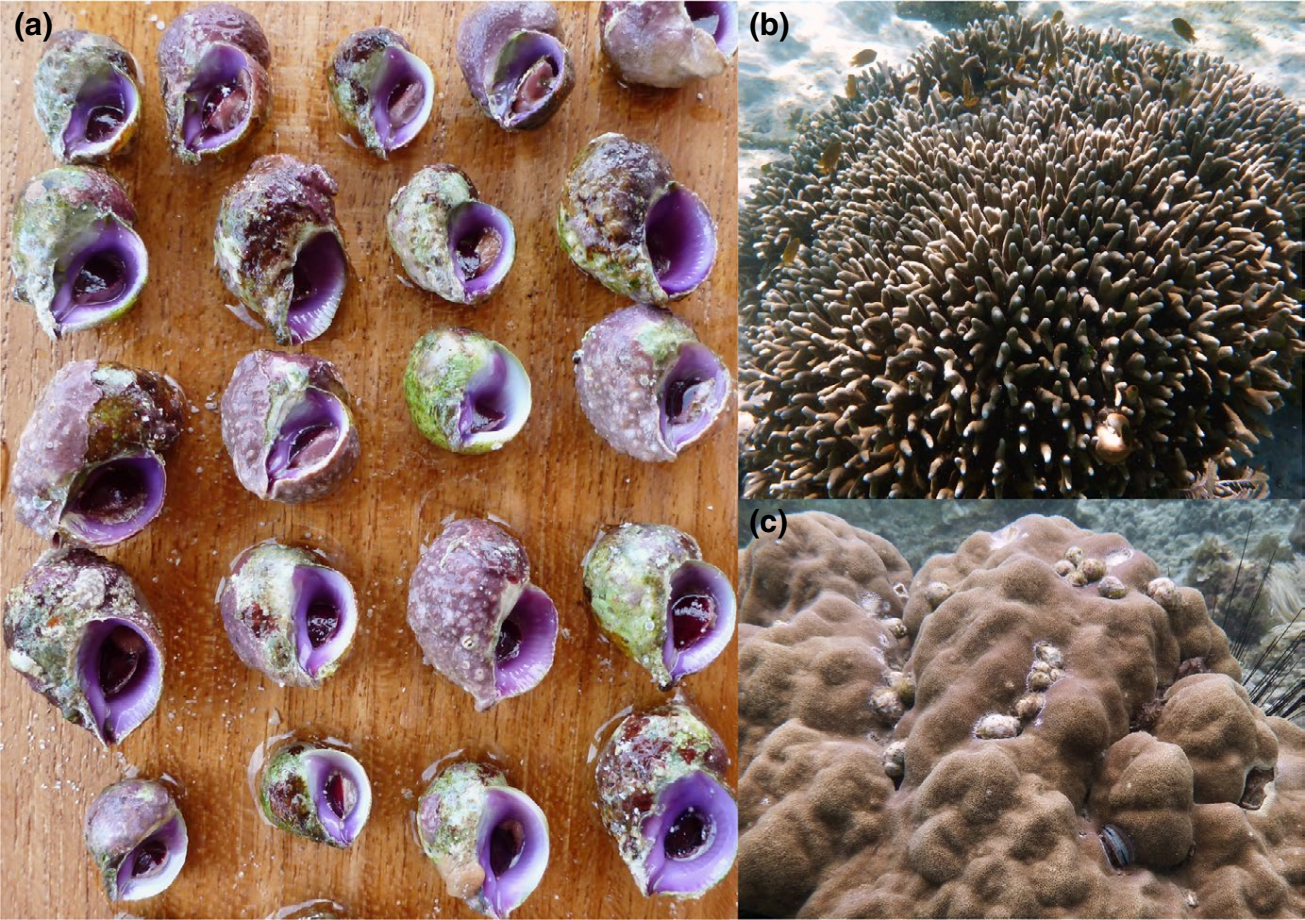
Violet coral snails, (a)* Coralliophila violacea* (Kiener, 1836), are obligate ectoparasites of corals in the family Poritidae. Their shells are usually fouled with crustose coralline algae because of their sedentary lifestyle, making them difficult to spot on their host corals. They are commonly found living among the branches of species such as (b) *Porites cylindrica* and can form aggregations on massive coral species like (c) *P. lobata*. (Photos by S.E. Simmonds)

The marine snail, *Coralliophila violacea* (Figure 1), is an obligate ectoparasite, living, feeding, and reproducing exclusively on corals in Poritidae, a highly abundant and diverse coral family (Kitahara, Cairns, Stolarski, Blair, & Miller, 2010), which is found in shallow reefs across the tropical Indo‐Pacific. The snails attach themselves to their host, form feeding aggregations, and drain energy from their host as it tries to repair damaged tissues (Oren, Brickner, & Loya, 1998). They are sequential hermaphrodites, a common trait of parasitic mollusks (Heller, 1993), and breed with conspecifics on their host coral colony. Two genetically distinct lineages of *C. violacea* occur sympatrically on reefs of the Coral Triangle, but each lineage occupies one of two groups of *Porites* corals, suggesting ecological divergence (Simmonds et al., 2018). A lack of evidence of genetic structure within each lineage of *C. violacea* inside the Coral Triangle precludes physical isolation as an explanation for the observed divergence. Host specificity commonly results from preferential larval settlement (Ritson‐Williams, Shjegstad, & Paul, 2003, 2007, 2009). This genetic evidence combined with observations of adult preference for specific coral hosts (unpubl. data S. Simmonds) strongly suggests ecological divergence driven by host association.

To determine where diverging populations of *C. violacea* lie on the continuum of the speciation process (i.e., host‐associated lineages, sibling species or good species), it is important to examine patterns of realized gene flow between the divergent coral host‐associated lineages. Effective contemporary gene flow should result in linkage disequilibria between host‐associated marker loci in populations utilizing different hosts. However, if lower rates of gene flow (<1% per generation) are found, then populations should be considered incipient species (Drès & Mallet, 2002; Malausa et al., 2007).

Genomic tests of selection are key to distinguishing between these possibilities. If divergence among *C. violacea* lineages results purely from neutral processes, genetic drift and migration should have approximately equal effects on all parts of the genome (Nielsen, 2005), and frequencies of neutral loci should show similar levels of differentiation (Via, 2009). However, if divergent selection is driving diversification of *C. violacea* lineages, there should be clear signatures of divergent selection (Feder et al., 1994; Nosil, Funk, & Ortiz‐Barrientos, 2009), because natural selection affects non‐neutral parts of the genome, as well as linked loci, to a greater extent (Smith & Haigh, 1974). As such, frequencies of loci under selection (outlier loci) or linked loci should either be unusually high or unusually low, in host‐associated populations, depending on the type of selection occurring (Beaumont & Nichols, 1996).

In this study, we use genome‐wide single nucleotide polymorphisms (SNPs) to investigate the possibility of ecological divergence with gene flow in populations of a corallivorous gastropod, *C. violacea*, from the Coral Triangle. Specifically, we (a) test for reduced gene flow between sympatric lineages of host‐associated snails, (b) identify outlier loci under putative selection between hosts, and (c) annotate possible functions of linked genes that might be necessary for adaptation to hosts.

## 2 MATERIALS AND METHODS

### 2.1 Sample collection

**Figure 2:**
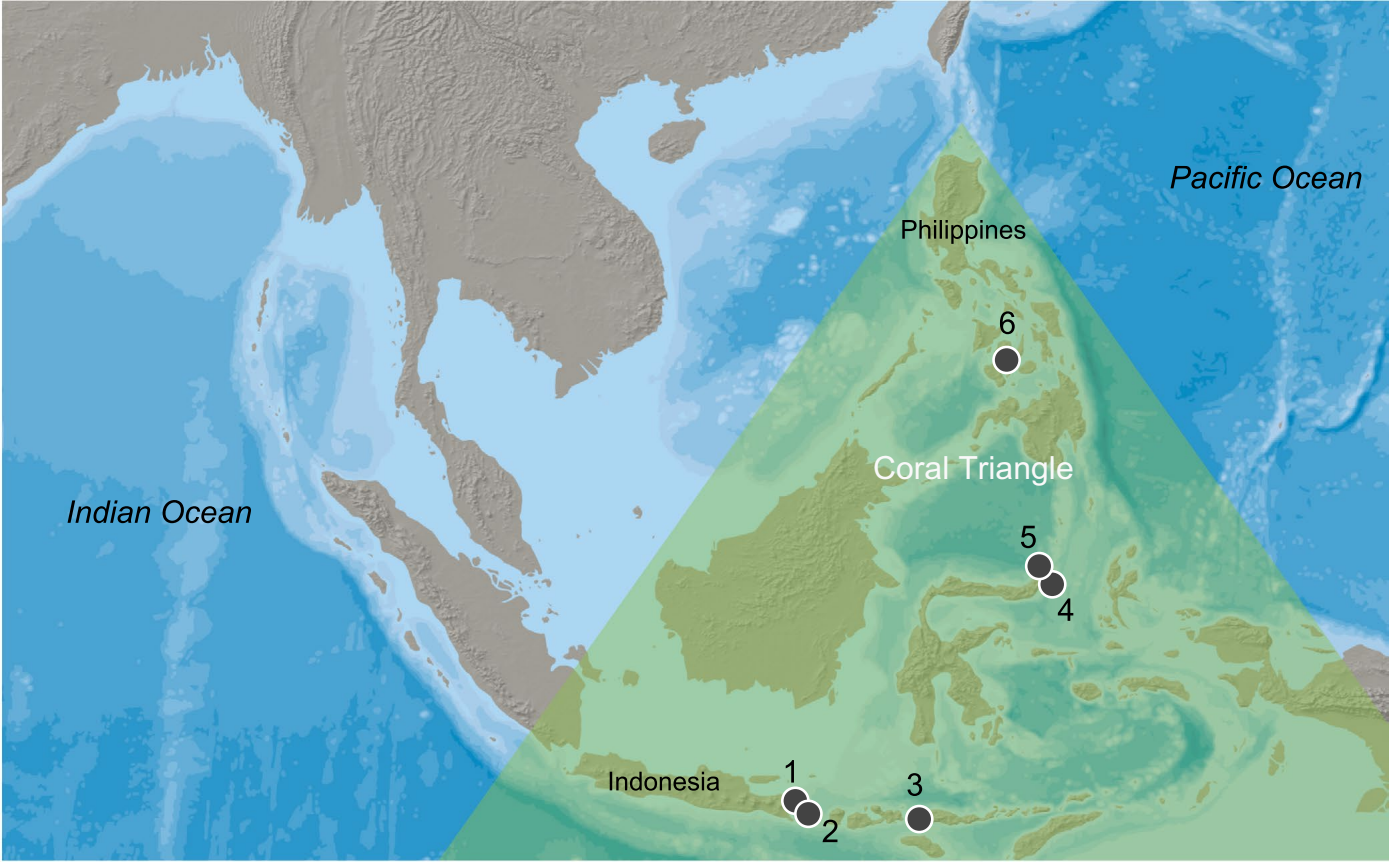
Collection locations for *Coralliophila violacea* from coral host species *Porites lobata* and *P. cylindrica*. 1. Pemuteran, 2. Nusa Penida, 3. Pulau Mengyatan, 4. Lembeh, 5. Bunaken, 6. Dumaguete. Map made with vector and raster map data available at http://naturalearthdata.com

We collected snails on snorkel during 2011–2013 from six sympatric populations of two lineages of *C. violacea* representing unique parasite–host groups (Table 1, Figure 2, Appendix S1). We chose snails from the most abundant *Porites* species from each group (*P. lobata*, *P. cylindrica*, Dana, 1846, Figure 1) to maximize the number of samples and reduce potentially confounding effects of differences among hosts within the same group. To further reduce confounding effects resulting from taxonomic complexity within *P. lobata* (Forsman, Barshis, Hunter, & Toonen, 2009; Prada et al., 2014), we used coral host species identifications from Simmonds et al. (2018) that were confirmed through RAD‐seq data.

**Table 1:** *Coralliophila violacea* collection locations, latitude, longitude, coral host species, and number of samples collected

Location	Country	Province	Latitude	Longitude	Coral host species	
					*Porites lobata*	*Porites cylindrica*
1. Pemuteran	Indonesia	Bali	−8.1400	114.6540	–	7
2. Nusa Penida	Indonesia	Bali	−8.6750	115.5130	11	10
3. Pulau Mengyatan	Indonesia	East Nusa Tenggara	−8.5570	119.6850	4	3
4. Lembeh	Indonesia	North Sulawesi	1.4790	125.2510	7	1
5. Bunaken	Indonesia	North Sulawesi	1.6120	124.7830	9	6
6. Dumaguete	Philippines	Negros Oriental	9.3320	123.3120	2	7
				Total *N*	33	34

### 2.2 Creation of RAD libraries

We extracted genomic DNA from 20 mg of foot tissue from 67 individual *C. violacea* (34 from *P. cylindrica* and 33 from *P. lobata*; Table 1) using a DNeasy^®^ Blood and Tissue Kit (QIAGEN), following manufacturer's instructions, save for elution of DNA with molecular grade H_2_O rather than AE buffer. We estimated initial DNA concentrations using a NanoDrop^™^ 2000 Spectrophotometer (Thermo Scientific^™^) and visualized DNA quality on a 1% agarose gel stained with SYBR^®^ Safe DNA Gel Stain (Invitrogen^™^). We used only high‐quality DNA with a bright high molecular weight band and minimal smearing. We dried DNA extractions using a SpeedVac^™^ (Thermo Scientific^™^) on medium heat and reconstituted using molecular grade H_2_O to a final uniform 250 ng/µl DNA concentration.

We created reduced representation libraries to survey SNP variation following published protocols (Wang, Meyer, McKay, & Matz, 2012) as updated by Dr. Eli Meyer (http://people.oregonstate.edu/~meyere/docs/Preparing2bRAD.pdf). AlfI restriction enzyme digest reduced representation (1/16th) libraries were labeled with individual barcodes and subjected to 18–20 PCR amplification cycles. The number of PCR cycles varied based on the optimal number determined in the test‐scale PCR to find the minimum number of cycles to produce a visible product at 166 bp. We electrophoresed products on a 2% agarose gel in 1 × TBE buffer and ran at 150 V for 90 min, visualized target bands (165 bp) with SYBR^®^ Safe DNA Gel Stain (Invitrogen^™^), and excised them from the gel. Then, we purified the excised bands using a QIAquick^®^ Gel Extraction Kit (QIAGEN). A final cleaning step used Agencourt^®^ AMPure^®^ XP beads (Beckman Coulter). QB3 Genomics at the University of California, Berkeley performed quality checks (qPCR, BioAnalyzer) and sequencing, multiplexing 10–20 snails per lane in 5 lanes of a 50 bp Single‐End run on an Illumina HiSeq 2000 sequencer.

### 2.3 RAD‐seq data processing

To prepare raw sequence data for SNP identification, we truncated all raw sequence reads to the insert size (36 bp), filtered for quality (PHRED scores >20), and discarded empty constructs. We then processed the resulting data using custom scripts written by Misha Matz, available on the 2bRAD GitHub site (https://github.com/z0on/2bRAD_denovo). First, we counted unique tag sequences (minimum sequencing depth 5×) and the number of sequences in reverse‐complement orientation and then merged these tags into one table. Then, we clustered all sequences in CD‐HIT (Fu, Niu, Zhu, Wu, & Li, 2012) using a 91% similarity threshold. Next, we defined the most abundant sequence in the cluster as a reference sequence and then filtered a locus‐annotated table from the previous two steps, excluding reads below 5× depth and those exhibiting strand bias. Lastly, we flipped the orientation of the resulting clustered sequences to match the most abundant tag in a cluster.

To call genotypes (as population‐wide RAD‐tag haplotypes), we used GATK (McKenna et al., 2010) and applied mild allele filters (10× total depth, allele bias cutoff 10, and strand bias cutoff 10), with the additional requirement that alleles appear in at least two individuals. We then applied locus filters allowing a maximum of 50% heterozygotes at a locus, no more than two alleles, genotyped in 30% of samples and polymorphic. Finally, we removed loci with the fraction of heterozygotes >75% (potential lumped paralogs) and missing >70% of genotypes. The final set of SNPs was then thinned to one per tag (that with the highest minor allele frequency) for *F*_ST_ and STRUCTURE analysis to remove linked loci.

### 2.4 Individual sample filtering steps

To maximize the quality of the final dataset, we further filtered out individuals (*N* = 11) with low genotyping rates, indicating low DNA quality, by taking the log_10_ of the number of sites genotyped per individual, and removing any individuals that were outside one standard deviation (*SD*) of the mean. We used VCFtools (Danecek et al., 2011) to estimate inbreeding coefficients and removed individuals (*N* = 5) with inbreeding coefficients outside the normal range (±2 *SD* of mean *F*) indicating possible low coverage sequencing or lumped paralogs (https://github.com/z0on/2bRAD_denovo). The remaining 51 individuals were used in analyses of population genetic structure. The final data file was in VCF format and converted to other formats using PGDSpider v2.0.8.0 (Lischer & Excoffier, 2012).

### 2.5 Genetic structure

To test whether the patterns observed in a mitochondrial locus were present in loci genome‐wide, we inferred the population genetic structure of the full RAD‐seq dataset (2,718 loci), outlier loci only (73 loci), and neutral loci only (2,645 loci), from 51 individuals using two methods. First, we ran the Bayesian model‐based clustering method STRUCTURE (Pritchard, Stephens, & Donnelly, 2000) using a burn‐in period of 20,000 followed by 50,000 MCMC replicates for *K* = 1–12, and 10 runs for each *K*. We used the admixture model, with allele frequencies correlated among populations. The results from STRUCTURE were then analyzed in CLUMPAK v1.1 (Kopelman, Mayzel, Jakobsson, Rosenberg, & Mayrose, 2015) to select for the best *K* and graphically display the results.

### 2.6 Outlier analyses

To test for evidence of natural selection in relation to coral host, we compared SNPs between lineages of snails on different hosts, pooled across six localities, with two datasets: (a) including all individuals and (b) excluding migrants and admixed individuals that we identified using STRUCTURE. First, we performed an outlier loci analysis using BayeScan v2.1 (Foll & Gaggiotti, 2008) with a prior of 10, a sample size of 5,000, and 100,000 iterations, using a burn‐in of 50,000, and 20 pilot runs of 5,000 each. To explore the impact of misleading data, we employed a 10% false discovery rate.

To further explore outlier loci, we used a second method to detect loci under selection (FDIST2) as implemented in ARLEQUIN (Excoffier & Lischer, 2010). We ran 100 demes per group and 50 groups for 50,000 simulations. This model compares a simulated neutral distribution of *F*_ST_ to the observed distribution and identifies outliers. Loci with significant *F*_ST_ *p* values (<0.01) were considered to be under selection (Excoffier & Lischer, 2010).

### 2.7 Candidate gene identification and annotation

To annotate the putative functions of genes linked to outlier loci, we aligned sequences containing SNP outlier loci to nucleotide collections (nr/nt) available on the NCBI website, in Blast2GO 5 Basic version (October 7, 2019) using the BLASTn algorithm (Altschul et al., 1997) with a taxonomic filter for Mollusca (taxid:6447). We adjusted parameters (expected threshold 10, word size 7, no low complexity filter, no mask for look‐up table only) to accommodate short read sequences. We only examined hits with a high query coverage (>80%). Then, we identified and annotated any associated genes using NCBI and GeneCards^®^.

## 3 RESULTS

After removing empty constructs and filtering for quality, we obtained an average of 5,710,091 unique sequence reads per individual at a minimum 5× depth. In total, we sequenced and genotyped 17,676 high‐quality RAD‐seq loci with ≥25× coverage, in 67 snails collected from two different coral host species, at six locations. After filtering for 30% maximum missing data per locus, this total decreased to 5,999 loci and then to 2,718 SNPs following thinning to one SNP per loci to remove any physically linked SNPs for STRUCTURE and *F*_ST_ analyses. Next, we removed 16 individuals that had either low DNA quality (missing data ≥ +1SD from the mean) or potential contamination issues (inbreeding coefficient ≥ +2SD from the mean), leaving 51 individuals.

### 3.1 Genetic structure

**Figure 3:**
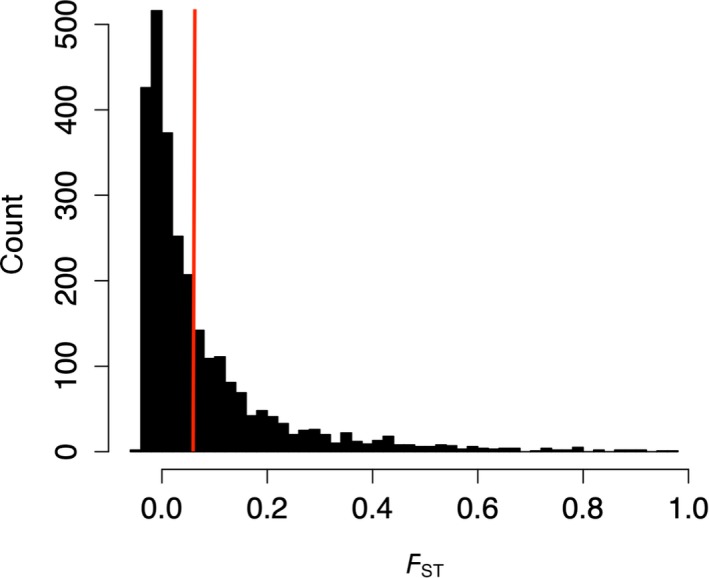
Histogram of variation in *F*_ST_ between lineages of *Coralliophila violacea* on two different coral hosts (*Porites lobata* and *P. cylindrica*) across all SNPs, excluding migrants and admixed individuals. *F*_ST_ calculated using FDIST in ARLEQUIN. Red line indicates the mean *F*_ST_ value (0.075)

**Figure 4:**
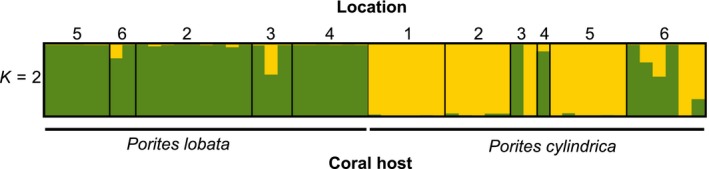
Bar plot of Bayesian assignment probability from STRUCTURE for *K* = 2 using 2,718 loci from 51 *Coralliophila violacea*. Each vertical bar corresponds to an individual. The proportion of each bar represents an individual's assignment probability to cluster one (green) or two (gold), shown grouped by coral host and then by location as numbered in Table 1, Figure 2

Tests of genetic differentiation between sympatric snail lineages on different coral hosts revealed moderate but significant structure (mean *F*_ST_ = 0.047, weighted *F*_ST_ = 0.090 (Weir & Cockerham, 1984)), between host‐associated lineages of snails (Figure 3). CLUMPAK analysis of the STRUCTURE results indicated *K* = 2 as the best *K* value (Appendix S2). At *K* = 2, the majority (88%) of all snails grouped by their coral host (Figure 4). Grouping by host was stronger in snails collected from *P. lobata* (97%) than from *P. cylindrica* (79%). Neutral loci (2,645) and outlier loci only (73) showed similar patterns of population structure in STRUCTURE to the full dataset of SNPs (Appendix S3).

### 3.2 Migration and admixture

Inferring the ancestry of individuals in STRUCTURE, using host as a prior, revealed strong differences among *C. violacea* living on different coral hosts (*P. lobata* and *P. cylindrica*, Figure 4), despite some migration and admixing between sympatric lineages. Moreover, migration rates were strongly asymmetric between snails living on these two hosts. In total, 19% (5 of 26 samples) of the snails collected from *P. cylindrica* had *P. lobata* genetic ancestry, while no snails (0 of 25 samples) with *P. cylindrica* ancestry were ever found on *P. lobata* (Appendix S4 and S5). Admixed individuals were only found at locations where migration was also observed (Dumaguete and Pulau Mengyatan; Appendix S5). After excluding migrants and admixed individuals, the mean *F*_ST_ across all loci increased from 0.047 to 0.075 and the weighted *F*_ST_ from 0.090 to 0.150.

### 3.3 Host‐specific directional selection

**Figure 5:**
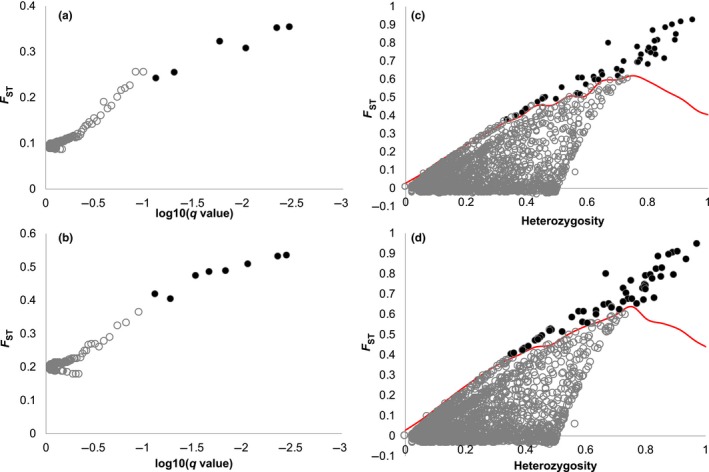
(a)–(b). Results from BayeScan analysis of full RAD‐seq dataset (2,718 loci) from *Coralliophila violacea*. Filled gray dots are *F*_ST_ outlier loci. (a) All individuals, 6 outlier loci identified FDR = 0.10, (b) excluding migrants and admixed individuals, 8 outlier loci identified FDR = 0.10. (c)–(d). Results from FDIST2 analysis implemented in ARELQUIN using the hierarchical island model of migration. Full RAD‐seq dataset (2,718 loci) from *Coralliophila violacea*. Filled black dots are *F*_ST_ outlier loci above the 99% quantile (red line). (c) All individuals, 51 outliers, (d) excluding migrants and admixed individuals, 65 outliers

Because STRUCTURE identified 9/51 individuals that were either migrants from one coral host to the other, or of admixed ancestry (Appendix S5), we used two different datasets for detecting host‐specific selection: (a) all individuals in the filtered dataset and (b) excluding migrants and admixed individuals. We then searched for loci under selection using two methods. The first involved a Bayesian model, BayeScan (Foll & Gaggiotti, 2008). Using the default false discovery rate (FDR) of 10%, we identified six loci as outliers (pairwise *F*_ST_ = 0.241–0.354, mean *F*_ST_ = 0.305, Figure 5a, Table 2) in the dataset with all snails. Three of these outlier loci (tag21753, tag39884, tag52997) had log_10_ (PO)> 1 giving substantial‐to‐strong support as candidate loci, based on criteria from (Jeffreys, 1961). After excluding all admixed and migrant individuals, the number of outlier loci only increased to eight (pairwise *F*_ST_ = 0.419–0.543, mean *F*_ST_ = 0.480, Figure 5b, Table 2). Four of these outlier loci (tag21753, tag28478, tag39884, and tag25141) had log_10_ (PO)> 1 giving substantial‐to‐strong support as candidate loci, based on criteria from (Jeffreys, 1961). All outlier loci had positive alpha values, indicating they are under directional selection between snails on different coral hosts.

**Table 2:** Outlier loci analysis from *Coralliophila violacea* found on different coral hosts (*Porites lobata*, *P. cylindrica*), BLAST hits, and functional annotations

Outlier analysis					BLAST search results								Gene ontology	
Dataset	Method	FST	log10(PO)	Tag ID	DNA sequence	Organism	Description	Score	Coverage (%)	E‐value	Identity (%)	Gene symbol	GO terms	Predicted function
all ind.	FDIST2	0.716		21753	AGGTCCTCTCTGGCACTGAGCTGCCAAGCTTCCACA	*Mizuhopecten yessoensis*	Prosaposin‐like	35.6	80%	0.23	86%	*PSAPL1*	Lipid metabolic process	NA
all ind.	Bayescan	0.354	2.465										Adenylate cyclase‐inhibiting G protein‐coupled receptor signaling pathway	
no mig./adm.	FDIST2	0.885											Sphingolipid metabolic process	
no mig./adm.	Bayescan	0.474	1.125										Regulation of metabolic process	
all ind.	FDIST2	0.665		28478	CATCCCCTCTATGCAACAGTATGCAAGTCCCCCTCT									
all ind.	Bayescan	0.241	0.585											
no mig./adm.	FDIST2	0.948												
no mig./adm.	Bayescan	0.534	2.446											
all ind.	FDIST2	0.718		39884	GGGTTGGCTGTAGCAACCTGCTGCCCCCAAAACCTT									
all ind.	Bayescan	0.3511	2.2244											
no mig./adm.	FDIST2	0.905												
no mig./adm.	Bayescan	0.484	1.2823											
all ind.	FDIST2	0.659	1.743	52997	CCAGGGATCAGCAGTCTCCTGCCACTGTTCCACAAG	*Aplysia californica*	Hemocyanin 1	34.6	86%	0.81	84%	*KLH1*	Metal‐ion binding	NA
no mig./adm.	FDIST2	0.91											Oxidoreductase activity	
all ind.	Bayescan	0.507												
all ind.	FDIST2	0.654	1.456	25141	GGCTAAAAAGTTGCATTGCTGTGCACAAAAAGTTCA									
no mig./adm.	FDIST2	0.869												
no mig./adm.	Bayescan	0.488												
all ind.	FDIST2	0.633		14249	AGACAAATTGCCGCACACACATGCAGACAAAACACA	*Aplysia californica*	Histone–lysine *N*‐methyltransferase 2D‐like	38.3	80%	0.066	90%	*KMT2D*	Metal‐ion binding	NA
													Methyltransferase activity	
all ind.	Bayescan	0.321	1.378										Transcription coactivator activity	
no mig./adm.	FDIST2	0.798											DNA binding	
all ind.	FDIST2	0.702		19628	GGCTATGGGTTTGCAAGGGAGTGCACTCTGCAATCA									
no mig./adm.	FDIST2	0.893												
no mig./adm.	Bayescan	0.403	0.603											
all ind.	FDIST2	0.54		36127	TGATCAAGCTTCGCATCGGTCTGCGCTCTCTTCTTC									
no mig./adm.	FDIST2	0.869												
no mig./adm.	Bayescan	0.419	0.508											
all ind.	FDIST2	0.588		30631	AGCAAGAGAATTGCACAAGGATGCGACCACAGAATG									
no mig./adm.	FDIST2	0.83												
all ind.	FDIST2	0.65		37258	GATGATCCTGCAGCAGTGTACTGCCTCTCTCTCTCT	*Lottia gigantea*	Hypothetical protein	36.5	100%	0.23	84%	Hypothetical protein	NA	NA
no mig./adm.	FDIST2	0.823												
all ind.	FDIST2	0.478		10161	CACCCCCTCTATGCAACAATATGCACGTCCCCCTCT									
no mig./adm.	FDIST2	0.795												
all ind.	FDIST2	0.627		30668	AGCTGCTCTCTAGCAGGTGACTGCATGTTGTGTACG									
no mig./adm.	FDIST2	0.794												
all ind.	FDIST2	0.461		21640	AGCCTGGATACTGCAGTAACCTGCTTTACAGGAGCA									
no mig./adm.	FDIST2	0.788												
all ind.	FDIST2	0.515		24247	AGTTGCGGCAGGGCAGACTACTGCATTGACGATCCC									
no mig./adm.	FDIST2	0.784												
all ind.	FDIST2	0.572		38182	CGACGGCTAGTGGCAATGCTTTGCAATCGAACATCA	*Lottia gigantea*	Hypothetical protein	32.8	83%	2.8	83%	Hypothetical	NA	NA
no mig./adm.	FDIST2	0.775												
all ind.	FDIST2	0.55		17358	CAGAATGTTCATGCAGTCCCATGCCATGTCTCAACT	*Mizuhopecten yessoensis*	Uncharacterized	37.4	83%	0.066	87%	Uncharacterized	NA	NA
no mig./adm.	FDIST2	0.768												
all ind.	FDIST2	0.541		38553	AGCACACGACATGCATTTCTGTGCCTGAGAAATGCC									
no mig./adm.	FDIST2	0.742												
all ind.	FDIST2	0.485		33555	AGGCCTTCATCAGCATCCCAGTGCATCTCAGGAACA									
no mig./adm.	FDIST2	0.735												
all ind.	FDIST2	0.518		22329	TGCTAACACAAGGCATAGTATTGCGACATATAACCG	*Crassostrea gigas*	Uncharacterized	38.3	91%	0.066	85%	Uncharacterized	NA	NA
no mig./adm.	FDIST2	0.729												
all ind.	FDIST2	0.536		21872	CGACTCGCGAATGCATTCTTTTGCTGCCTCTTTTTC									
no mig./adm.	FDIST2	0.727												
all ind.	FDIST2	0.456		39420	TGTTTGGCTATGGCAGCTGTGTGCTACAACAGAATT									
no mig./adm.	FDIST2	0.721												
all ind.	FDIST2	0.468		33550	TGAGGAAACACAGCATTAGTTTGCAAATTTATTTCT									
no mig./adm.	FDIST2	0.705												
all ind.	FDIST2	0.415		30176	AGGCCTTTTATGGCAAACAGCTGCAACATACTGCCA									
no mig./adm.	FDIST2	0.679												
all ind.	FDIST2	0.526		32580	CACCGTTATCTGGCACAACAGTGCGACGCCTGAACT									
no mig./adm.	FDIST2	0.673												
all ind.	FDIST2	0.525		28305	TGCTTGCAACATGCACGCATATGCACACCACAAACT									
no mig./adm.	FDIST2	0.67												
all ind.	FDIST2	0.471		10755	GGTGTGAAATTGGCAGGCAAATGCCTTACTCATCCT									
no mig./adm.	FDIST2	0.659												
all ind.	FDIST2	0.498		24085	GGATAAAAGCGCGCACCAAAATGCGCATAATTTTCT	*Pomacea canaliculata*	PR domain zinc finger protein 8‐like	30.1	86%	9.9	81%	*PRDM8*	Metal‐ion binding	NA
													Histone methyltransferase activity	
no mig./adm.	FDIST2	0.652											Chromatin binding	
all ind.	FDIST2	0.462		32708	TGTGATACTCTTGCACTTTACTGCAAAGGCCATGTT	*Octopus bimaculoides*	AP2‐associated protein kinase 1‐like	35.6	91%	0.23	85%	*AAK1*	Kinase, serine/threonine‐protein kinase, transferase	NA
no mig./adm.	FDIST2	0.646											DNA binding, ATP binding, endocytosis	
all ind.	FDIST2	0.57		24158	GGCCTGATCACTGCAGGATCTTGCTGGTATTTGTCA									
no mig./adm.	FDIST2	0.634												
all ind.	FDIST2	0.429		28347	AGAAAAAGAGGCAGAGAAAGATATGGGAGAAGAACA	*Aplysia californica*	Nuclear hormone receptor HR96‐like	39.2	100%	0.019	83%	*HR96*	Metal‐ion binding	Xenobiotic detoxification
													DNA binding	
													Receptor	
no mig./adm.	FDIST2	0.617												
all ind.	FDIST2	0.417		37421	AACTCAAAAATCGCATTTGTTTGCTTTAGTTGCGCT									
no mig./adm.	FDIST2	0.614												
all ind.	FDIST2	0.463		22275	TGCAATTGCGAAGCAAATGTCTGCTCTGGTGCGCCG									
no mig./adm.	FDIST2	0.611												
all ind.	FDIST2	0.404		24087	TGCATATTGTGTGCAGTGCCTTGCAGAGTATATGCC									
no mig./adm.	FDIST2	0.599												
all ind.	FDIST2	0.427		16452	AGTGACTGGAGAGCACTTGTTTGCGGCCTATGTTCC	*Littorina saxatilis*	NA	41	88%	0.005	88%	Uncharacterized		
no mig./adm.	FDIST2	0.587												
all ind.	FDIST2	0.432		27928	CGTGACAACGCCGCAACAGAGTGCCTTGGGGACGCC									
no mig./adm.	FDIST2	0.557												
all ind.	FDIST2	0.458		48048	GACACGACAACTGCAGCCAGTTGCTTCCCTTGATCG									
no mig./adm.	FDIST2	0.556												
all ind.	FDIST2	0.414		17029	TGGTGTTACCTTGCAGTCAACTGCATTTATTCCTCT									
no mig./adm.	FDIST2	0.554												
all ind.	FDIST2	0.374		34705	AGCAGTCTCACTGCAGTTTTCTGCACTGCATAAACT									
no mig./adm.	FDIST2	0.526												
all ind.	FDIST2	0.34		20904	TGGCAAGACCTGGCAAACAGCTGCTGAGATGGGACC									
no mig./adm.	FDIST2	0.522												
all ind.	FDIST2	0.372		20142	AGATTCATGCCAGCACAATCCTGCAAGACACTATCC									
no mig./adm.	FDIST2	0.52												
all ind.	FDIST2	0.388		21098	TGAGAAAAAGTTGCATGTGAGTGCGTGCATGGCGCG									
no mig./adm.	FDIST2	0.516												
all ind.	FDIST2	0.334		27266	TGCAATGAAAACACATAAAAACACCTGTGTGCACTC									
no mig./adm.	FDIST2	0.471												
all ind.	FDIST2	0.407		15079	GGCTGAGCAGAGGCAGACGGCTGCGGAGCAGGAGGA	*Pomacea canaliculata*	Sodium‐dependent proline transporter‐like	43.7	86%	0.002	90%	*SLC6A7*	Neurotransmitter	Gastropod feeding behavior
no mig./adm.	FDIST2	0.451											Sodium symporter activity	
no mig./adm.	FDIST2	0.748		42043	CGCAATCGTATTGCAAAATTGTGCAATTGCTCCACT									
no mig./adm.	FDIST2	0.676		31609	CGAACAGATGTGGCAAAAGACTGCTGCCTTGGACCA									
no mig./adm.	FDIST2	0.651		22586	AGAGACAGAGTTGCATCCCTTTGCGTCGCACTCACC	*Octopus vulgaris*	Uncharacterized	30.1	100%	9.9	78%	Uncharacterized	NA	NA
no mig./adm.	FDIST2	0.636		22561	TGTGTGTGTGTTGCACCTACATGCACCTAAGTTACG									
no mig./adm.	FDIST2	0.624		31557	CGGAGGTTTGTAGCAGAGCCTTGCCTGCCATAGTCT	*Aplysia californica*	Neurogenic protein mastermind‐like	31.9	83%	2.8	87%	*MAM*	Developmental protein, neurogenesis, differentiation	NA
no mig./adm.	FDIST2	0.559		21042	AGGCTTTGAAGTGCATGCATGTGCAGCCGTCTGTCA									
no mig./adm.	FDIST2	0.555		33474	TGACACTAGTCAGCAGATAGATGCCAGGGATGGCCC									
no mig./adm.	FDIST2	0.514		11613	GGTCCGTGGCTTGCACAGGGATGCAATGCAATGTCT									
no mig./adm.	FDIST2	0.492		15069	TGAACATGTCCAGCACCCTTTTGCGCTAAAGAACCT									
no mig./adm.	FDIST2	0.486		18108	CACATCCATCTCGCATAGTTCTGCTGATCCAGAGCA	*Crassostrea gigas*	NA	39.2	86%	0.019	87%	Uncharacterized	NA	NA
no mig./adm.	FDIST2	0.478		27744	GAAGTTACACAAGCACTGCCATGCGTAAAAATGACT									
no mig./adm.	FDIST2	0.476		32951	TACCTTGGGTATGCAACCCGATGCCAAGACCAAGAT									
no mig./adm.	FDIST2	0.448		33996	CACGTCCTGACAGCACAAACCTGCACTGATGTCTCT									
no mig./adm.	FDIST2	0.44		16737	TGTGTTGTGTGTGCAGGTTCATGCAGCTGATTGGTG									
no mig./adm.	FDIST2	0.431		13930	AGGTGAAATAAAGCAATGAAATGCAGGGCCGTGTCA	*Pomacea canaliculata*	Protein draper‐like		91%	0.81	82%	*DRPR*	Transmembrane receptor, phagocytosis	Larval locomotory behavior
no mig./adm.	FDIST2	0.428		34999	GGATCTGTCTCTGCAAAAGCTTGCCTGCTGATCTTG									
no mig./adm.	FDIST2	0.424		27749	TGAGACGTTAACGCATACGGCTGCTTTTAAGTAGCC									
no mig./adm.	FDIST2	0.424		17800	TGTGCTTCCTTGGCAGAACCCTGCAAAAATAATCTG									
no mig./adm.	FDIST2	0.407		13296	AGAAAATTCTTGGCACTGTGCTGCTATTGCTTATCA									
no mig./adm.	FDIST2	0.404		17181	AGCACACAGCACGCACGTGTTTGCACACCAAGAGCA									
no mig./adm.	FDIST2	0.373		16929	GGGTAATCCAAAGCAACTCAGTGCCTTACCCCCCCT									
no mig./adm.	FDIST2	−0.033		23096	CACCCCCTCTATGCAAAGTCATGCAAGTCTGCCTCT									
all ind.	FDIST2	0.638		21172	GGTACTAAAAAAGCAACCGTATGCGTAATCGTCTCA									
all ind.	Bayescan	0.255	0.655											
all ind.	FDIST2	0.593		20062	CACCATGTCTATGCACGTGCATGCAGACACTGGGCA									
all ind.	FDIST2	0.491		38482	AGGGCACACAGGGCACACAGATGCACATCTTACTCA									
all ind.	FDIST2	0.417		32340	GAGTTGTCCAAGGCAAAATTCTGCAGAAAGGAAACA									
all ind.	FDIST2	0.366		33003	TGAGGCTATTTTGCATGCAGCTGCTAGATCTCTTCC									
all ind.	FDIST2	0.323		9230	TGCAAGCTTTTTGCATTCCTTTGCAAATCGAAGGCT									
all ind.	FDIST2	0.225		19533	TGCTCATTACTCGCATACTGTTGCTCTGTTCAGACT									
all ind.	FDIST2	0.195		11006	CGCAGAAGGAAGGCAAGCAGATGCCTAATAATCGCT									

Note

Only the results that met cutoff statistics are shown.

Abbreviations: adm., admixed; ind., individuals; mig., migrants.

In the second method, FDIST2, we used the infinite island model of migration to identify 51 outlier loci (pairwise *F*_ST_ = 0.177–0.729, mean *F*_ST_ = 0.492, Figure 5c) in the dataset with all snails. After removing migrants and admixed individuals, the number of outliers increased to 65 with higher *F*_ST_ values (pairwise *F*_ST_ = 0.320–0.925, mean *F*_ST_ = 0.620, Figure 5d) indicating directional selection, resulting in a combined total of 73 outlier loci across the two methods and datasets. Of these 73, a total of 43 outlier loci were shared between the two datasets; 8 were unique to the all‐individual dataset, and 22 were unique to the dataset that excluded migrants and admixed individuals (Table 2). Three outlier loci (tag28478, tag21753, and tag39884) were common among all datasets and methods (Table 2).

### 3.4 Mapping and annotation of outlier loci

The majority (78%) of putative outlier loci did not align to any other mollusk sequences currently available in the NCBI database (11/2019, Table 2). Sixteen outlier loci DNA sequences aligned with a variety of mollusks including four gastropods (*Aplysia californica, Littorina saxatilis*, *Lottia gigantea,* and *Pomacea canalicutata*), three bivalves (*Mizuhopecten yessoensis*, *Crassostrea gigas*, and *C. virginica*), and two cephalopods (*Octopus bimaculoides* and *O. vulgaris*) (Table 2). Of these loci, 7 mapped to hypothetical or uncharacterized proteins. The remaining 9 loci mapped to gene regions with predicted functions. The annotated genes had various associated gene ontology terms including lipid metabolism, metal‐ion binding, methyltransferase activity, immune response, chromatin binding, DNA binding, and serine/threonine‐protein kinase. The top two hits (lowest e‐values) were a neurotransmitter gene (tag15079, *SLC6A7* gene) that plays a role in gastropod feeding behavior (Miller, 2019), and a hormone receptor gene (tag28347, *HR96* gene) involved in the regulation of xenobiotic detoxification (Lindblom & Dodd, 2006; Richter & Fidler, 2014). At tag28347, there were two alleles that occurred in almost equal frequency (43%, 57%) in the *P. lobata*‐associated lineage of snails but were nearly fixed (97%) for one allele in the *P. cylindrica*‐associated lineage of snails. Another gene of interest (tag13930, *DRPR* gene) codes for receptors involved in larval locomotory behavior (Freeman, Delrow, Kim, Johnson, & Doe, 2003).

## 4 DISCUSSION

Genome‐wide SNP data from six sympatric populations of *C. violacea* revealed two clearly differentiated clusters that were largely concordant with coral host, consistent with results from mitochondrial DNA (Simmonds et al., 2018). As with insects (Jean & Jean‐Christophe, 2010; Simon et al., 2015), this genome‐wide differentiation supports the conclusion of ecological divergence based on host association and adds to a small but growing literature on ecological divergence in marine environments (Fritts‐Penniman et al., 2020; Potkamp & Fransen, 2019; Titus, Blischak, & Daly, 2019).

While SNP data reveal significant divergence between host‐specific lineages of *C. violacea,* divergence was substantially lower in genome‐wide SNPs compared to mtDNA (*F*_ST_ = 0.047 vs. Φ_CT_ = 0.561). This result may partially be a function of the smaller effective population size of the mitochondrial genome (Palumbi, Cipriano, & Hare, 2001). However, lower divergence values also suggest intermediate levels of gene flow between distinct host‐associated lineages (Nm>10), values that are similar to other cases of sympatric host‐associated divergence (e.g., Gouin et al., 2017; Peccoud, Ollivier, Plantegenest, & Simon, 2009; Smadja et al., 2012). Divergence with gene flow is further supported by the presence of admixed genotypes and unidirectional gene flow from one host lineage to the other. Moreover, considerable detection of outlier loci under directional selection (2.7% of all SNP loci; 73/2,718) strongly suggests that selection by coral host is likely contributing to the partitioning of *C. violacea* lineages.

### 4.1 Divergence with gene flow

In parasitic species such as *C. violacea*, divergence with gene flow likely occurs through two mechanisms of premating isolation (Nosil, Vines, & Funk, 2005). The first is host preference for egg laying and/or recruitment to their host (either individual or species). Divergence occurs when mating takes place solely on that host, eventually leading to speciation (Funk, Filchak, & Feder, 2002; Hawthorne & Via, 2001). Second is host adaptation, where selection acts against immigrants from another host via immigrant inviability (Nosil, 2007; Nosil et al., 2005; Porter & Benkman, 2017). Our study suggests that both mechanisms may be occurring in *C. violacea*. All migrants were individuals that genetically sorted to the lineage associated with *P. lobata* but were instead living on *P. cylindrica*. Additionally, only admixed individuals were observed on *P. lobata*. This pattern suggests that gene flow and admixture between host‐associated lineages are unidirectional—from *lobata* to *cylindrica*. Such unidirectional gene flow could result from two possible scenarios, either the failure of larvae to recruit, or the failure of recruited larvae to survive.

Larval recruitment processes could promote asymmetrical gene flow if the lineage associated with *P. cylindrica* strongly prefers *P. cylindrica* as a host over *P. lobata* or does not respond to chemical settlement cues from *P. lobata,* preventing the recruitment of *P. cylindrica‐*associated larvae to *P. lobata*. In addition, larvae from *P. lobata* would need to be less selective in their recruitment, occasionally landing on *P. cylindrica* rather than *P. lobata*. Such a mechanism makes sense, given that there are twice as many coral species (*N* = 8) in the clade of *Porites* to which *P. lobata* belongs, than in that to which *P. cylindrica* belongs.

An alternative, but not mutually exclusive explanation is that asymmetry in gene flow and admixture could result from postsettlement processes. For example, if larvae from *P. cylindrica*‐associated individuals settle on *P. lobata,* but are less likely to survive and reproduce, this could lead to immigrant inviability (Ingley & Johnson, 2016; Nosil et al., 2005; Richards & Ortiz‐Barrientos, 2016) and asymmetry in admixture. Under such a scenario, genes beneficial to snails living on *P. cylindrica* would likely be less helpful on *P. lobata* and we should see some indication of a selective sweep in the derived lineage with respect to the standing genetic variation of the ancestral lineage (Przeworski, Coop, & Wall, 2005). Indeed, results showed some outlier loci (e.g., *HR96*, detoxification gene) that were in equal proportions in *P. lobata* (43%, 57%) but were at near fixation in *P. cylindrica* (97%), indicating a soft sweep on standing genetic variation at that locus.

Regardless of whether the limited misalignment of snails and coral hosts results from pre‐ or postrecruitment processes, the fact that the vast majority of snails sort by host coral in the face of hybridization and gene flow indicates that natural selection must be relatively strong to counteract gene flow of Nm>10 (Funk, Egan, & Nosil, 2011). Moreover, the high fidelity of the snails occupying *P. cylindrica* and lower fidelity of snails occupying *P. lobata*, combined with selective sweeps in *P. cylindrica,* suggest that snails parasitizing *P. lobata* are the ancestral lineage. This conjecture is consistent with the observation that specialist species often evolve from generalist ancestors (Nosil, 2002), likely because specialization constrains further evolution by reducing genetic variation (Moran, 1988). If it is generally true that specialists evolve from generalists (Kawecki, 1996, 1998), then host specialization could be an important mechanism of divergence within the Coral Triangle (Briggs, 2005) as increased diversity should raise niche partitioning, leading to more opportunities for host specialization (Janz, Nylin, & Wahlberg, 2006).

### 4.2 Candidate genes involved in adaptation to host

Outlier loci can provide insights into the targets of natural selection (Storz, 2005) and are a useful starting point for determining how selection may be acting on lineages diverging on different hosts. Our analysis revealed 73 putative gene regions with *F*_ST_ values significantly higher than neutral expectations, suggesting that they are likely under selection and could be involved in adaptation to coral hosts, or linked to such genes via hitchhiking (Via, 2012).

There is no a priori information on the types of genes involved in the adaptation of mollusks to different hosts and, due to a lack of genomic resources for *C. violacea*, only 9 of 73 outlier loci mapped to gene regions with predicted functions. However, a useful comparison can be found in ectoparasitic phloem‐feeding insects adapting to different host plants (Oren et al., 1998). Genes under selection in these insect–plant interactions include genes involved in sensing hosts, that protect insects against plant defenses and facilitate feeding, and that code for digestive and detoxifying enzymes to neutralize plant toxins (e.g., metal‐ion binding, Simon et al., 2015).

Experimental evidence suggests genes with metal‐ion binding functions are repeatedly under selection in stick insects adapting to different host plants (Soria‐Carrasco et al., 2014). Indeed, four of the *C. violacea* candidate genes we identified in outlier tests are involved in metal‐ion binding (*KTM2D*, *KLH1*, *PRDM8*, and *HR96*). Very little is known about how corals and their algal symbionts chemically defend themselves against or react to parasites and predators. *Symbiodinium* species do produce toxins—Zooxanthellatoxins—(Gordon & Leggat, 2010), but it is unknown whether these toxins are upregulated in response to parasites or predators.

Additional evidence for detoxification playing a role in host divergence comes from *HR96,* a nuclear hormone receptor involved in xenobiotic detoxification (Richter & Fidler, 2014). Interestingly, *HR96* was nearly fixed for one allele in *C. violacea* from *P. cylindrica* (97%) but was at 50% in *C. violacea* from *P. lobata*, which indicates a selective sweep at that locus. This result, combined with the four metal‐ion binding gene regions, suggests that there may be important differences in host‐associated detoxification processes in the different *C. violacea* lineages. If adaptation to host‐specific toxins drives host specificity, mismatches between snail metabolic abilities and coral hosts could explain the strong asymmetry in snails being found on an atypical coral host.

While the above results suggest a putative detoxification role for some outlier loci, two other genes with predicted functions, a neurotransmitter (*SLC6A7*) important for gastropod feeding behavior (Miller, 2019) and a transmembrane receptor (*DRPR*) involved in larval locomotory behavior, indicate a possible role of behavior in adaptation (Freeman et al., 2003). Notably, this is only the first genomic exploration of *C. violacea* and a broader survey of genomic diversity would be needed to pin down areas of the genome that are crucial for adaptations to coral hosts. Future work would benefit from a fully annotated genome of *C. violacea* that would allow us to examine the genomic architecture of divergence with gene flow and quantitative trait loci. In turn, this would allow us to better pinpoint regions of the genome under selection, and the specific functions of genes involved in adapting to different hosts.

### 4.3 Ecological divergence in the sea

John Briggs originally proposed the idea of sympatric speciation as an important diversification mechanism within the Coral Triangle (i.e., “Center of Origin” hypothesis), as well as in the export of species formed under intense competition within the region (Briggs, 1999, 2005). To support his hypothesis, he pointed to multiple cases of sympatric sibling species with distributions centered on the Coral Triangle, where the older of the two species has a wide range, while the younger has a much more restricted range limited to the Coral Triangle (Briggs, 1999). Our study provides the first genomic evidence to support his assertion that ecological divergence with gene flow could be generating biodiversity in the Coral Triangle. In addition, spatial patterning of *C. violacea* sympatric host lineages also matches the pattern Briggs described, with the ancestral *P. lobata* host lineage having a broad geographic distribution, and the derived *P. cylindrica* host lineage restricted to the Coral Triangle (Simmonds et al., 2018).

As the global epicenter of marine biodiversity, there is a large and diverse literature on the processes shaping the Coral Triangle (Barber, Erdmann, & Palumbi, 2006; Bowen et al., 2013; Carpenter et al., 2011; Gaither et al., 2011; Hoeksema, 2007; Kochzius & Nuryanto, 2008; Tornabene, Valdez, Erdmann, & Pezold, 2015). While there is ongoing debate (Evans, McKenna, Simpson, Tournois, & Genner, 2016; Huang, Goldberg, Chou, & Roy, 2018; Di Martino, Jackson, Taylor, & Johnson, 2018; Matias & Riginos, 2018), there is clearly a multiplicity of processes driving diversification in this region (Barber & Meyer, 2015). Given the results of this study, it is important to expand our thinking beyond models that focus solely on allopatry to advance our understanding of marine speciation and origins of the Coral Triangle biodiversity hotspot.

## AUTHOR CONTRIBUTIONS

SES conceived of and designed the study. SES, AFP, and SHC collected samples, prepared libraries, and analyzed genomic data. All authors worked on and approved of the manuscript.

## Data Availability

Raw single‐end Illumina HiSeq 2000 reads and RAD‐seq loci datasets are archived on Dryad: https://doi.org/10.5068/D1995V.
