# Supplementary material for: Genomic signatures of host‐associated divergence and adaptation in a coral‐eating snail, Coralliophila violacea (Kiener, 1836)
Source: Ecol Evol. 2020 Feb 5;10(4):1817–37. doi: 10.1002/ece3.5977 (PMC7042750; doi:10.1002/ece3.5977)
Supplement: Supplementary file 1 [file ECE3-10-1817-s001.docx]

**Appendices for:**

**Genomic signatures of host-associated divergence and**

**adaptation in a coral-eating snail, *Coralliophila violacea***

**(Kiener, 1836)**

Sara E. Simmonds^1*^, Allison L. Fritts-Penniman^1^, Samantha H. Cheng^1^, G. Ngurah Mahardika^2^ & Paul H. Barber^1^

**Appendix S1.** All the collection and sequencing information for samples collected in this study. Raw reads are available on Dryad: <https://doi.org/10.5068/D1995V>

| Sample ID | Organism | Location | Country | Latitude | Longitude | Year | Host | Sequence ID | QC Filter |
| --- | --- | --- | --- | --- | --- | --- | --- | --- | --- |
| ID2003.01 | *Coralliophila violacea* | 1. Pemuteran | Indonesia | -8.1400 | 114.6540 | 2011 | *Porites cylindrica* | SSPB003A |  |
| ID2003.05 | *Coralliophila violacea* | 1. Pemuteran | Indonesia | -8.1400 | 114.6540 | 2011 | *Porites cylindrica* | SSPB003B |  |
| ID2003.07 | *Coralliophila violacea* | 1. Pemuteran | Indonesia | -8.1400 | 114.6540 | 2011 | *Porites cylindrica* | SSPB003C |  |
| ID2004.05 | *Coralliophila violacea* | 1. Pemuteran | Indonesia | -8.1400 | 114.6540 | 2011 | *Porites cylindrica* | SSPB003D | Removed |
| ID2004.07 | *Coralliophila violacea* | 1. Pemuteran | Indonesia | -8.1400 | 114.6540 | 2011 | *Porites cylindrica* | SSPB003E |  |
| ID2004.08 | *Coralliophila violacea* | 1. Pemuteran | Indonesia | -8.1400 | 114.6540 | 2011 | *Porites cylindrica* | SSPB003F |  |
| ID2005.01 | *Coralliophila violacea* | 1. Pemuteran | Indonesia | -8.1400 | 114.6540 | 2011 | *Porites cylindrica* | SSPB004N |  |
| ID2124.02 | *Coralliophila violacea* | 2. Nusa Penida | Indonesia | -8.6750 | 115.5130 | 2012 | *Porites cylindrica* | SSPB003S |  |
| ID2126.02 | *Coralliophila violacea* | 2. Nusa Penida | Indonesia | -8.6750 | 115.5130 | 2012 | *Porites cylindrica* | SSPB003V |  |
| ID2124.01 | *Coralliophila violacea* | 2. Nusa Penida | Indonesia | -8.6750 | 115.5130 | 2012 | *Porites cylindrica* | SSPB004AA | Removed |
| ID2124.03 | *Coralliophila violacea* | 2. Nusa Penida | Indonesia | -8.6750 | 115.5130 | 2012 | *Porites cylindrica* | SSPB004CC | Removed |
| ID2122.01 | *Coralliophila violacea* | 2. Nusa Penida | Indonesia | -8.6750 | 115.5130 | 2012 | *Porites cylindrica* | SSPB004X | Removed |
| ID2122.02 | *Coralliophila violacea* | 2. Nusa Penida | Indonesia | -8.6750 | 115.5130 | 2012 | *Porites cylindrica* | SSPB004Y |  |
| ID2123.03 | *Coralliophila violacea* | 2. Nusa Penida | Indonesia | -8.6750 | 115.5130 | 2012 | *Porites cylindrica* | SSPB004Z | Removed |
| ID2123.04 | *Coralliophila violacea* | 2. Nusa Penida | Indonesia | -8.6750 | 115.5130 | 2012 | *Porites cylindrica* | SSPB005O |  |
| ID2126.01 | *Coralliophila violacea* | 2. Nusa Penida | Indonesia | -8.6750 | 115.5130 | 2012 | *Porites cylindrica* | SSPB005S | Removed |
| ID2123.01 | *Coralliophila violacea* | 2. Nusa Penida | Indonesia | -8.6750 | 115.5130 | 2012 | *Porites cylindrica* | SSPB005N |  |
| ID2104.02 | *Coralliophila violacea* | 3. Pulau Mengyatan | Indonesia | -8.5570 | 119.6850 | 2012 | *Porites cylindrica* | SSPB002T |  |
| ID2110.03 | *Coralliophila violacea* | 3. Pulau Mengyatan | Indonesia | -8.5570 | 119.6850 | 2012 | *Porites cylindrica* | SSPB003Q |  |
| ID2105.03 | *Coralliophila violacea* | 3. Pulau Mengyatan | Indonesia | -8.5570 | 119.6850 | 2012 | *Porites cylindrica* | SSPB004S | Removed |
| ID2072.03 | *Coralliophila violacea* | 4. Lembeh | Indonesia | 1.4790 | 125.2510 | 2012 | *Porites cylindrica* | SSPB002Z |  |
| ID2089.01 | *Coralliophila violacea* | 5. Bunaken | Indonesia | 1.6120 | 124.7830 | 2012 | *Porites cylindrica* | SSPB003M |  |
| ID2089.02 | *Coralliophila violacea* | 5. Bunaken | Indonesia | 1.6120 | 124.7830 | 2012 | *Porites cylindrica* | SSPB003N |  |
| ID2089.05 | *Coralliophila violacea* | 5. Bunaken | Indonesia | 1.6120 | 124.7830 | 2012 | *Porites cylindrica* | SSPB003O |  |
| ID2089.10 | *Coralliophila violacea* | 5. Bunaken | Indonesia | 1.6120 | 124.7830 | 2012 | *Porites cylindrica* | SSPB003P |  |
| ID2089.06 | *Coralliophila violacea* | 5. Bunaken | Indonesia | 1.6120 | 124.7830 | 2012 | *Porites cylindrica* | SSPB005D |  |
| ID2102.04 | *Coralliophila violacea* | 5. Bunaken | Indonesia | 1.6120 | 124.7830 | 2012 | *Porites cylindrica* | SSPB002R |  |
| PH0003.06 | *Coralliophila violacea* | 6. Dumaguete | Philippines | 9.3320 | 123.3120 | 2013 | *Porites cylindrica* | SSPB002B |  |
| PH0009.13 | *Coralliophila violacea* | 6. Dumaguete | Philippines | 9.3320 | 123.3120 | 2013 | *Porites cylindrica* | SSPB002BB |  |
| PH0003.09 | *Coralliophila violacea* | 6. Dumaguete | Philippines | 9.3320 | 123.3120 | 2013 | *Porites cylindrica* | SSPB002Q | Removed |
| PH0009.03 | *Coralliophila violacea* | 6. Dumaguete | Philippines | 9.3320 | 123.3120 | 2013 | *Porites cylindrica* | SSPB002QQ |  |
| PH0009.01 | *Coralliophila violacea* | 6. Dumaguete | Philippines | 9.3320 | 123.3120 | 2013 | *Porites cylindrica* | SSPB002U |  |
| PH0003.08 | *Coralliophila violacea* | 6. Dumaguete | Philippines | 9.3320 | 123.3120 | 2013 | *Porites cylindrica* | SSPB002W |  |
| PH0003.04 | *Coralliophila violacea* | 6. Dumaguete | Philippines | 9.3320 | 123.3120 | 2013 | *Porites cylindrica* | SSPB001B |  |
| ID2121.06 | *Coralliophila violacea* | 2. Nusa Penida | Indonesia | -8.6750 | 115.5130 | 2012 | *Porites lobata* | SSPB003R |  |
| ID2125.07 | *Coralliophila violacea* | 2. Nusa Penida | Indonesia | -8.6750 | 115.5130 | 2012 | *Porites lobata* | SSPB003T |  |
| ID2125.01 | *Coralliophila violacea* | 2. Nusa Penida | Indonesia | -8.6750 | 115.5130 | 2012 | *Porites lobata* | SSPB004DD |  |
| ID2125.02 | *Coralliophila violacea* | 2. Nusa Penida | Indonesia | -8.6750 | 115.5130 | 2012 | *Porites lobata* | SSPB004EE |  |
| ID2121.05 | *Coralliophila violacea* | 2. Nusa Penida | Indonesia | -8.6750 | 115.5130 | 2012 | *Porites lobata* | SSPB004U |  |
| ID2121.07 | *Coralliophila violacea* | 2. Nusa Penida | Indonesia | -8.6750 | 115.5130 | 2012 | *Porites lobata* | SSPB004V |  |
| ID2121.08 | *Coralliophila violacea* | 2. Nusa Penida | Indonesia | -8.6750 | 115.5130 | 2012 | *Porites lobata* | SSPB004W |  |
| ID2121.02 | *Coralliophila violacea* | 2. Nusa Penida | Indonesia | -8.6750 | 115.5130 | 2012 | *Porites lobata* | SSPB005L | Removed |
| ID2121.03 | *Coralliophila violacea* | 2. Nusa Penida | Indonesia | -8.6750 | 115.5130 | 2012 | *Porites lobata* | SSPB005M |  |
| ID2125.05 | *Coralliophila violacea* | 2. Nusa Penida | Indonesia | -8.6750 | 115.5130 | 2012 | *Porites lobata* | SSPB005P | Removed |
| ID2125.09 | *Coralliophila violacea* | 2. Nusa Penida | Indonesia | -8.6750 | 115.5130 | 2012 | *Porites lobata* | SSPB005Q |  |
| ID2108.01 | *Coralliophila violacea* | 3. Pulau Mengyatan | Indonesia | -8.5570 | 119.6850 | 2012 | *Porites lobata* | SSPB002C |  |
| ID2114.01 | *Coralliophila violacea* | 3. Pulau Mengyatan | Indonesia | -8.5570 | 119.6850 | 2012 | *Porites lobata* | SSPB002CC | Removed |
| ID2120.06 | *Coralliophila violacea* | 3. Pulau Mengyatan | Indonesia | -8.5570 | 119.6850 | 2012 | *Porites lobata* | SSPB002RR |  |
| ID2120.09 | *Coralliophila violacea* | 3. Pulau Mengyatan | Indonesia | -8.5570 | 119.6850 | 2012 | *Porites lobata* | SSPB004T |  |
| ID2081.07 | *Coralliophila violacea* | 4. Lembeh | Indonesia | 1.4790 | 125.2510 | 2012 | *Porites lobata* | SSPB004O |  |
| ID2081.04 | *Coralliophila violacea* | 4. Lembeh | Indonesia | 1.4790 | 125.2510 | 2012 | *Porites lobata* | SSPB003H |  |
| ID2081.08 | *Coralliophila violacea* | 4. Lembeh | Indonesia | 1.4790 | 125.2510 | 2012 | *Porites lobata* | SSPB003I | Removed |
| ID2083.03 | *Coralliophila violacea* | 4. Lembeh | Indonesia | 1.4790 | 125.2510 | 2012 | *Porites lobata* | SSPB003J |  |
| ID2083.04 | *Coralliophila violacea* | 4. Lembeh | Indonesia | 1.4790 | 125.2510 | 2012 | *Porites lobata* | SSPB003K |  |
| ID2081.02 | *Coralliophila violacea* | 4. Lembeh | Indonesia | 1.4790 | 125.2510 | 2012 | *Porites lobata* | SSPB003G |  |
| ID2083.05 | *Coralliophila violacea* | 4. Lembeh | Indonesia | 1.4790 | 125.2510 | 2012 | *Porites lobata* | SSPB005B |  |
| ID2084.04 | *Coralliophila violacea* | 5. Bunaken | Indonesia | 1.6120 | 124.7830 | 2012 | *Porites lobata* | SSPB004L |  |
| ID2101.02 | *Coralliophila violacea* | 5. Bunaken | Indonesia | 1.6120 | 124.7830 | 2012 | *Porites lobata* | SSPB004R | Removed |
| ID2084.02 | *Coralliophila violacea* | 5. Bunaken | Indonesia | 1.6120 | 124.7830 | 2012 | *Porites lobata* | SSPB005C |  |
| ID2093.03 | *Coralliophila violacea* | 5. Bunaken | Indonesia | 1.6120 | 124.7830 | 2012 | *Porites lobata* | SSPB005E | Removed |
| ID2093.05 | *Coralliophila violacea* | 5. Bunaken | Indonesia | 1.6120 | 124.7830 | 2012 | *Porites lobata* | SSPB005F |  |
| ID2093.06 | *Coralliophila violacea* | 5. Bunaken | Indonesia | 1.6120 | 124.7830 | 2012 | *Porites lobata* | SSPB005G |  |
| ID2094.05 | *Coralliophila violacea* | 5. Bunaken | Indonesia | 1.6120 | 124.7830 | 2012 | *Porites lobata* | SSPB005I |  |
| ID2094.06 | *Coralliophila violacea* | 5. Bunaken | Indonesia | 1.6120 | 124.7830 | 2012 | *Porites lobata* | SSPB005J | Removed |
| ID2101.05 | *Coralliophila violacea* | 5. Bunaken | Indonesia | 1.6120 | 124.7830 | 2012 | *Porites lobata* | SSPB002DD | Removed |
| PH0001.02 | *Coralliophila violacea* | 6. Dumaguete | Philippines | 9.3320 | 123.3120 | 2013 | *Porites lobata* | SSPB001F |  |
| PH0004.01 | *Coralliophila violacea* | 6. Dumaguete | Philippines | 9.3320 | 123.3120 | 2013 | *Porites lobata* | SSPB002HH |  |

**a) b)**


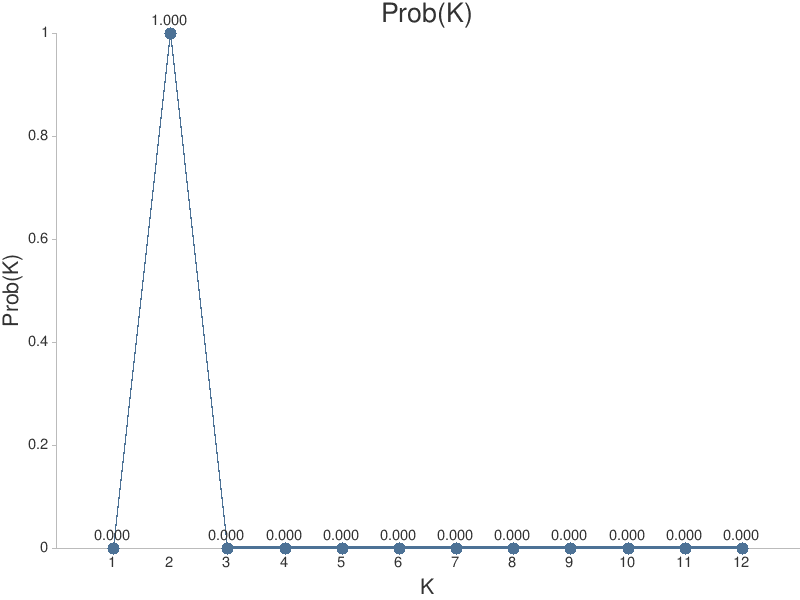

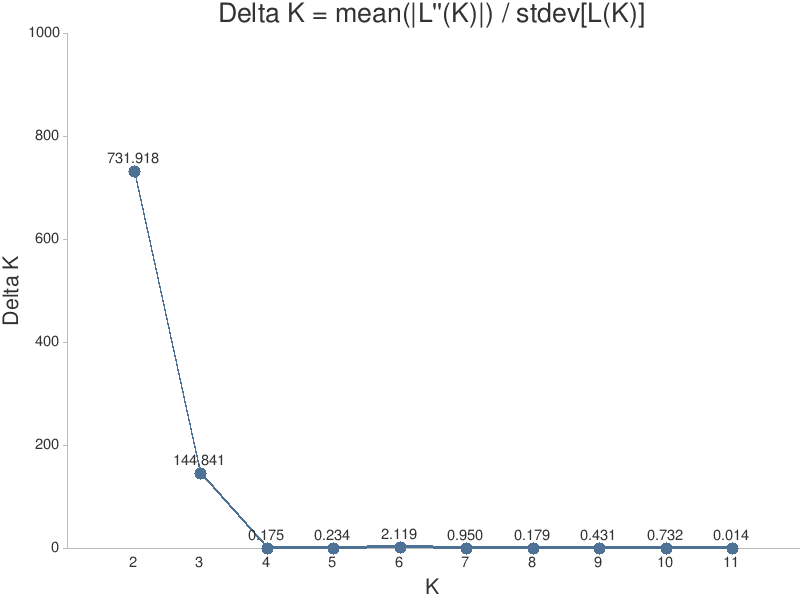


**Appendix S2.** Estimates of the best *K* using **a)** the Evanno method (Evanno et al., 2005), and **b)** the method from STRUCTURE identifying the *K* with the highest Pr(*K*=k). Both methods indicate *K* = 2 is best.


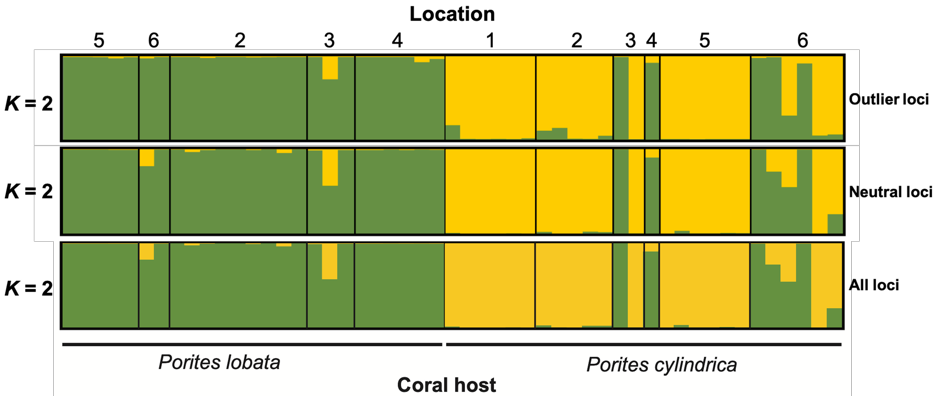


**Appendix S3.** Bar plot of Bayesian assignment probability from STRUCTURE for *K* = 2 using all 2,718 loci, 2, from 51 *Coralliophila violacea*. Each vertical bar corresponds to an individual. The proportion of each bar represents an individual’s assignment probability to cluster one (green) or two (gold), shown grouped by coral host and then by location as numbered in Table 1.

**Appendix S4.** *Coralliophila violacea*. Full RAD-seq dataset of 2,718 loci from all individuals, grouped by coral host. STRUCTURE results for *K* = 2.

|  |  | | Inferred cluster | |
| --- | --- | --- | --- | --- |
| Location | Coral host | *P. cylindrica* | | *P. lobata* |
| 1. Pemuteran | *P. cylindrica* | 98% | | 2% |
| 1. Pemuteran | *P. cylindrica* | 100% | | 0% |
| 1. Pemuteran | *P. cylindrica* | 100% | | 0% |
| 1. Pemuteran | *P. cylindrica* | 100% | | 0% |
| 1. Pemuteran | *P. cylindrica* | 100% | | 0% |
| 1. Pemuteran | *P. cylindrica* | 100% | | 0% |
| 2. Nusa Penida | *P. cylindrica* | 97% | | 3% |
| 2. Nusa Penida | *P. cylindrica* | 100% | | 0% |
| 2. Nusa Penida | *P. cylindrica* | 100% | | 0% |
| 2. Nusa Penida | *P. cylindrica* | 98% | | 2% |
| 2. Nusa Penida | *P. cylindrica* | 98% | | 2% |
| 3. Pulau Mengyatan | *P. cylindrica* | 0% | | 100% |
| 3. Pulau Mengyatan | *P. cylindrica* | 100% | | 0% |
| 4. Lembeh | *P. cylindrica* | 10% | | 90% |
| 5. Bunaken | *P. cylindrica* | 100% | | 0% |
| 5. Bunaken | *P. cylindrica* | 97% | | 3% |
| 5. Bunaken | *P. cylindrica* | 100% | | 0% |
| 5. Bunaken | *P. cylindrica* | 100% | | 0% |
| 5. Bunaken | *P. cylindrica* | 99% | | 1% |
| 5. Bunaken | *P. cylindrica* | 100% | | 0% |
| 6. Dumaguete | *P. cylindrica* | 0% | | 100% |
| 6. Dumaguete | *P. cylindrica* | 25% | | 75% |
| 6. Dumaguete | *P. cylindrica* | 45% | | 55% |
| 6. Dumaguete | *P. cylindrica* | 0% | | 100% |
| 6. Dumaguete | *P. cylindrica* | 100% | | 0% |
| 6. Dumaguete | *P. cylindrica* | 77% | | 23% |

| Location | Coral host | *P. cylindrica* | *P. lobata* |
| --- | --- | --- | --- |
| **2. Nusa Penida** | *P. lobata* | 0% | 100% |
| **2. Nusa Penida** | *P. lobata* | 2% | 98% |
| **2. Nusa Penida** | *P. lobata* | 1% | 99% |
| **2. Nusa Penida** | *P. lobata* | 0% | 100% |
| **2. Nusa Penida** | *P. lobata* | 0% | 100% |
| **2. Nusa Penida** | *P. lobata* | 1% | 99% |
| **2. Nusa Penida** | *P. lobata* | 0% | 100% |
| **2. Nusa Penida** | *P. lobata* | 3% | 97% |
| **2. Nusa Penida** | *P. lobata* | 0% | 100% |
| **3. Pulau Mengyatan** | *P. lobata* | 1% | 99% |
| **3. Pulau Mengyatan** | *P. lobata* | 42% | 58% |
| **3. Pulau Mengyatan** | *P. lobata* | 0% | 100% |
| **4. Lembeh** | *P. lobata* | 0% | 100% |
| **4. Lembeh** | *P. lobata* | 0% | 100% |
| **4. Lembeh** | *P. lobata* | 0% | 100% |
| **4. Lembeh** | *P. lobata* | 0% | 100% |
| **4. Lembeh** | *P. lobata* | 0% | 100% |
| **4. Lembeh** | *P. lobata* | 0% | 100% |
| **5. Bunaken** | *P. lobata* | 0% | 100% |
| **5. Bunaken** | *P. lobata* | 0% | 100% |
| **5. Bunaken** | *P. lobata* | 0% | 100% |
| **5. Bunaken** | *P. lobata* | 1% | 99% |
| **5. Bunaken** | *P. lobata* | 0% | 100% |
| **6. Dumaguete** | *P. lobata* | 19% | 81% |
| **6. Dumaguete** | *P. lobata* | 0% | 100% |

**Appendix S5**. *Coralliophila violacea* grouped by the coral host they were collected from as a prior, showing the probability of ancestry to host-associated lineages and if they had a parent or grandparent from the other host-associated lineage. We identified individuals classified as migrants* and as admixed** in STRUCTURE with the USESPOPINFO model.

|  |  | Probability of ancestry | | | |  |
| --- | --- | --- | --- | --- | --- | --- |
| Locality | Coral host | *P. cylindrica* | *P. lobata* | Parent | Grandparent | Classification |
| 1. Pemuteran | *P. cylindrica* | 100% | 0% | 0% | 0% |  |
| 1. Pemuteran | *P. cylindrica* | 100% | 0% | 0% | 0% |  |
| 1. Pemuteran | *P. cylindrica* | 100% | 0% | 0% | 0% |  |
| 1. Pemuteran | *P. cylindrica* | 100% | 0% | 0% | 0% |  |
| 1. Pemuteran | *P. cylindrica* | 100% | 0% | 0% | 0% |  |
| 1. Pemuteran | *P. cylindrica* | 100% | 0% | 0% | 0% |  |
| 2. Nusa Penida | *P. cylindrica* | 100% | 0% | 0% | 0% |  |
| 2. Nusa Penida | *P. cylindrica* | 100% | 0% | 0% | 0% |  |
| 2. Nusa Penida | *P. cylindrica* | 100% | 0% | 0% | 0% |  |
| 2. Nusa Penida | *P. cylindrica* | 100% | 0% | 0% | 0% |  |
| 2. Nusa Penida | *P. cylindrica* | 100% | 0% | 0% | 0% |  |
| 3. Pulau Mengyatan | *P. cylindrica* | 0% | 100% | 0% | 0% | migrant |
| 3. Pulau Mengyatan | *P. cylindrica* | 100% | 0% | 0% | 0% |  |
| 4. Lembeh | *P. cylindrica* | 0% | 100% | 0% | 0% | migrant |
| 5. Bunaken | *P. cylindrica* | 100% | 0% | 0% | 0% |  |
| 5. Bunaken | *P. cylindrica* | 100% | 0% | 0% | 0% |  |
| 5. Bunaken | *P. cylindrica* | 100% | 0% | 0% | 0% |  |
| 5. Bunaken | *P. cylindrica* | 100% | 0% | 0% | 0% |  |
| 5. Bunaken | *P. cylindrica* | 100% | 0% | 0% | 0% |  |
| 5. Bunaken | *P. cylindrica* | 100% | 0% | 0% | 0% |  |
| 6. Dumaguete | *P. cylindrica* | 0% | 100% | 0% | 0% | migrant |
| 6. Dumaguete | *P. cylindrica* | 0% | 100% | 0% | 0% | migrant |
| 6. Dumaguete | *P. cylindrica* | 0% | 8% | 22% | 70% | admixed |
| 6. Dumaguete | *P. cylindrica* | 0% | 100% | 0% | 0% | migrant |
| 6. Dumaguete | *P. cylindrica* | 100% | 0% | 0% | 0% |  |
| 6. Dumaguete | *P. cylindrica* | 0% | 0% | 0% | 100% | admixed |
|  |  |  |  |  |  |  |
|  |  | **Probability of ancestry** | | | |  |
| Location | **Coral host** | ***P. lobata*** | ***P. cylindrica*** | **Parent** | **Grandparent** | **Classification** |
| 2. Nusa Penida | *P. lobata* | 100% | 0% | 0% | 0% |  |
| 2. Nusa Penida | *P. lobata* | 100% | 0% | 0% | 0% |  |
| 2. Nusa Penida | *P. lobata* | 100% | 0% | 0% | 0% |  |
| 2. Nusa Penida | *P. lobata* | 100% | 0% | 0% | 0% |  |
| 2. Nusa Penida | *P. lobata* | 100% | 0% | 0% | 0% |  |
| 2. Nusa Penida | *P. lobata* | 100% | 0% | 0% | 0% |  |
| 2. Nusa Penida | *P. lobata* | 100% | 0% | 0% | 0% |  |
| 2. Nusa Penida | *P. lobata* | 100% | 0% | 0% | 0% |  |
| 2. Nusa Penida | *P. lobata* | 100% | 0% | 0% | 0% |  |
| 3. Pulau Mengyatan | *P. lobata* | 100% | 0% | 0% | 0% |  |
| 3. Pulau Mengyatan | *P. lobata* | 0% | 0% | 0% | 100% | admixed |
| 3. Pulau Mengyatan | *P. lobata* | 100% | 0% | 0% | 0% |  |
| 4. Lembeh | *P. lobata* | 100% | 0% | 0% | 0% |  |
| 4. Lembeh | *P. lobata* | 100% | 0% | 0% | 0% |  |
| 4. Lembeh | *P. lobata* | 100% | 0% | 0% | 0% |  |
| 4. Lembeh | *P. lobata* | 100% | 0% | 0% | 0% |  |
| 4. Lembeh | *P. lobata* | 100% | 0% | 0% | 0% |  |
| 4. Lembeh | *P. lobata* | 100% | 0% | 0% | 0% |  |
| 5. Bunaken | *P. lobata* | 100% | 0% | 0% | 0% |  |
| 5. Bunaken | *P. lobata* | 100% | 0% | 0% | 0% |  |
| 5. Bunaken | *P. lobata* | 100% | 0% | 0% | 0% |  |
| 5. Bunaken | *P. lobata* | 100% | 0% | 0% | 0% |  |
| 5. Bunaken | *P. lobata* | 100% | 0% | 0% | 0% |  |
| 6. Dumaguete | *P. lobata* | 0% | 0% | 0% | 100% | admixed |
| 6. Dumaguete | *P. lobata* | 100% | 0% | 0% | 0% |  |
|  |  |  |  |  |  |  |
